# Characteristics and correlation of gray matter volume and somatic symptoms in adolescent patients with depressive disorder

**DOI:** 10.3389/fpsyt.2023.1197854

**Published:** 2023-07-25

**Authors:** Daming Mo, Pengfei Guo, Shuwen Hu, Rui Tao, Hui Zhong, Huanzhong Liu

**Affiliations:** ^1^Department of Psychiatry, Chao hu Hospital of Anhui Medical University, Hefei, China; ^2^Department of Child and Adolescent Mental Disorder, Affiliated Psychological Hospital of Anhui Medical University, Hefei, China; ^3^Department of Psychiatry, Anhui Mental Health Center, Hefei, China; ^4^Department of Psychiatry, School of Mental Health and Psychological Sciences, Anhui Medical University, Hefei, China; ^5^Department of Psychiatry, Hangzhou Seventh People’s Hospital, Hangzhou, China; ^6^Clinical Psychological Science, Anhui Provincial Children’s Hospital, Hefei, China

**Keywords:** somatic symptoms, gray matter volume, alexithymia, adolescent, depressive disorder

## Abstract

**Background:**

Adolescent patients with depressive disorders commonly exhibit somatic symptoms, which have a significant negative impact on their treatment and prognosis. Despite this, specific brain imaging characteristics of these symptoms have been poorly studied.

**Methods:**

The Hamilton Depression Rating scale (HAMD-17), Children’s Functional Somatization scale (CSI), and Toronto Alexithymia scale (TAS) were used to evaluate the clinical symptoms of adolescent depression. We analyzed the correlation between brain gray matter volume (GMV) and clinical symptoms in adolescent patients with depression and somatic symptoms.

**Results:**

The depression subgroups with and without functional somatic symptoms (FSS) had higher scores on the HAMD-17, CSI, and TAS than the normal control group. The group with FSS had higher HAMD-17, CSI, and TAS scores than the depression group without FSS (*p* < 0.05). CSI and TAS scores were positively correlated (*r* = 0.378, *p* < 0.05). The GMV of the right supplementary motor area was higher in the depression groups with and without FSSs than in the normal control group, and the GMV was higher in the group without FSS than in the group with FSS (*F* = 29.394, *p* < 0.05). The GMV of the right supplementary motor area was negatively correlated with CSI in the depressed group with FSS (*r* = −0.376, *p* < 0.05). In the group with depression exhibiting FSS, CSI scores were positively correlated with GMV of the middle occipital gyrus (*pr* = 0.665, *p* = 0.0001), and TAS scores were positively correlated with GMV of the caudate nucleus (*pr* = 0.551, *p* = 0.001).

**Conclusion:**

Somatic symptoms of adolescent depressive disorder are associated with alexithymia; moreover, somatic symptoms and alexithymia in adolescent patients with depressive disorders are correlated with GMV changes in different brain regions.

## Introduction

Major depressive disorder (MDD) is one of the most common psychiatric disorders, and an epidemiological survey conducted in China in 2019 showed a lifetime prevalence of depressive disorder of 6.9% (1). The prevalence of depression is rising in lower age groups; an epidemiological survey conducted in China in 2022 among school students (aged 6–16 years) showed a prevalence of 3.2% for depressive disorders (2). Adolescent-onset depression has a high recurrence rate and symptom severity, as well as a high risk of suicide throughout the lifespan (3). This disorder severely affects patients’ social functioning, such as academic and interpersonal relationships (4). The clinical manifestations of depressive disorders are complex and diverse and mainly include three dimensions: affective, cognitive, and somatic symptoms (5). Studies have shown that approximately 70% of patients with depressive disorders exhibit somatic symptoms (6). Somatic symptoms are positively correlated with the severity of depression and negatively impact the course of depressive disorders (7). Functional somatic symptoms (FSS) are a group of somatic symptom clusters with unknown pathogenesis that mostly occur in children and adolescents (8). An epidemiological survey published in 2008 showed that 10–30% of children and adolescents in the United States have functional somatic symptoms (9). The detection rate of these symptoms among children and adolescents in urban Nanjing in China was 7.6% in 2018, with those in the lower age group showing more somatic symptoms than children in the higher age group and with boys showing more symptoms than girls (8).

A large body of research has been conducted to demonstrate the close relationship between somatic symptoms and mood disorders, such as depression and anxiety, in children. A 2012 study confirmed a clear correlation between depression and FSS (10). In the depressed child-adolescent population, somatic symptoms are reported twice as often as in the normal population (6). Somatic symptoms in childhood predict somatic and depressive symptoms, suicidal behavior, and other psychological problems in adulthood (11). Somatic symptoms are positively correlated with depression severity and suicidal ideation in patients with depression and may prolong the course of the disease (12). Moreover, somatic symptoms are a predictor of clinical outcomes in patients with depressive disorders; the more severe the symptoms, the worse the antidepressant efficacy (13). Studies have found that alexithymia is closely related to the somatic symptoms of depressive disorders (14) and that patients with depression have impairments related to sensory, comprehensive, and internal body signal integration (15). This misprediction of internal feelings affects the motivational behavior of individuals with depression, and the disconnection between these two processes can lead to somatic symptoms and alexithymia (16). However, the specific pathogenesis of depressive disorders with somatic symptoms in adolescents remains unclear.

Magnetic resonance imaging (MRI) studies of the brain have found that somatic symptoms of depressive disorders correspond to different brain regions. For example, fatigue is associated with brain regions such as the prefrontal cortex, striatum, and cerebellum (17), and sleep and appetite are associated with the hypothalamus and basal ganglia (18). Studies of somatic symptoms, internal perceptual abnormalities, and changes in brain function in patients with depressive disorders have found that dorsomedial insula activation is negatively correlated with somatic symptoms and depression severity (19), whereas one study found that somatic symptom scores were positively correlated with gray matter volume (GVM) of the left inferior frontal cortex and negatively correlated with cerebellar vermis and right supplementary motor area (Supp Motor Area-R) (20). Compared to patients with adulthood-onset depressive disorders, there was a relative increase in GMV in the early-onset depression group, namely in the medial prefrontal cortex, insular cortex, and anterior hippocampus (21). Clinically, the detection rate of somatic symptoms in adolescents is estimated to be between 25 and 75% (22), but only 5 to 7% of adolescents meet all the criteria for somatic disorders. Therefore, more appropriate scales are needed to assess the somatic symptoms of depression in adolescents (23).

Adolescence is a critical period of functional brain development that coincides with significant changes in physiological and social development. The maturation of the brain during adolescence includes both structural brain maturation and functional development (24); therefore, the somatic symptoms of adolescent depressive disorder may differ from those of adult depression. In summary, we hypothesized that somatic symptoms in adolescent depressive disorder may also be accompanied by corresponding structural brain abnormalities and investigated the neural mechanisms by examining the characteristics of cranial GMV in adolescent patients having depressive disorders with and without somatic symptoms and their correlation with clinical symptoms.

## Materials and methods

### Participants

This study consisted of 71 patients with adolescent depression who were hospitalized in the Department of Children and Adolescents of Anhui Mental Health Center, Hefei Fourth People’s Hospital, and 19 healthy participants. Patients with MDD were selected based on the following criteria: (1) meeting the diagnostic criteria for a major depressive episode in the Diagnostic and Statistical Manual of Mental Disorders, IV edition (DSM-IV); (2) Han nationality; and (3) age between 13 and 18 years. The exclusion criteria were as follows: (1) concomitant severe physical illness; (2) previous or current history of other DSM-IV axis I episodes; (3) previous history of severe alcohol and drug abuse; and (4) any contraindications for MRI.

The Children’s Somatization Inventory (CSI) was used to assess the presence of FSSs in the last 2 weeks after enrollment. Patients with adolescent depressive disorder were divided into two groups based on FSS presence (FSS group, *n* = 34 and non-FSS group, *n* = 37). The demographic data of the participants are presented in Table 1.

**Table 1:** Comparison of clinical data and clinical symptom scale scores among the three groups.

	HC (*n* = 19)	Non-FSS (*n* = 37)	FSS (*n* = 34)	*X*^2^/F/t	*P*
Sex(male/female)	4/15	7/30	8/26	0.226	0.893
Education	8.47 ± 1.78	8.21 ± 1.35	7.97 ± 1.46	0.785	0.459
Age	15.91 ± 1.92	15.21 ± 1.43	14.97 ± 1.54	2.437	0.093
Disease duration	/	18.18 ± 17.01	17.73 ± 13.79	1.23	0.903
HAMD_17_	4.65 ± 1.87^ab^	15.94 ± 7.48^c^	21.79 ± 6.99c	49.747	<0.001
CSI	4.65 ± 1.84^ab^	30.68 ± 15.30^c^	21.79 ± 6.99c	75.469	<0.001
TAS	44.95 ± 8.24^ab^	74.03 ± 10.01^c^	80.64 ± 9.89	100.756	0.001

^a^Was compared with the group without FSS, *p* < 0.01; ^b^was compared with the group with FSS, *p* < 0.01; ^c^Was compared with the FSS group, *p* < 0.01.

### General demographic data and clinical characteristic assessment scales

(1) Hamilton Depression Rating scale (HAMD-17) (25): This is a widely used clinical scale for the assessment of depressive symptoms. This version of the scale consists of 17 entries, including 5-factor scores for anxiety/somatization, cognitive impairment, weight, sleep disturbance, and blockage, and a final summary factor score, with higher scores indicating more severe depressive symptoms in the participant’s most recent week.
(2) The Children’s Somatization Inventory (CSI) (26): This involves the Functional Somatization Scale for Children, which was specifically designed to assess FSSs in children and adolescents. The Chinese version of this scale has 42 items, including four subfactors: gastrointestinal symptom, pain, cardiovascular symptom, and pseudoaneurysm. The score ranges between 0 and 168, where a score greater than 19 and an entry greater than or equal to 11 were considered FSS.
(3) The Toronto Alexithymia Scale (TAS) (27):It is a 26-item scale that includes four factors: the ability to describe emotions, the ability to recognize and distinguish emotions from somatic feelings, fantasy, and extroverted thinking.

### MRI data acquisition

MRI scans were obtained using a 3.0-Tesla MR system (Discovery MR750w; General Electric, Milwaukee, WI, United States) with a 16-channel head coil. Before scanning, after being informed of the testing procedure and contraindications by the MRI room staff, all participants were asked to close their eyes, be quiet and relaxed, remain awake but not actively think, use a tight but comfortable sponge pad to hold their head in place, and lay flat on the scanner with nano noise-canceling earplugs to reduce noise. The scanning sequence and parameters for obtaining high-resolution 3D T1-weighted 3D structural images are as follows: time of echo (TE) = 3.2 ms; time of repeat (TR) = 8.5 ms; flip angle (FA) = 12°; field of view (FOV) = 256 mm × 256 mm; matrix size = 256 × 256; slice thickness (ST) = 1 mm, no gap; voxel size = 1 mm × 1 mm × 1 mm; 188 sagittal slices; and scanning time of approximately 6 min. Resting-state BOLD fMRI data were acquired using a gradient echo single echo planar imaging (GRE-SS-EPI) sequence with the following parameters: TE = 30 ms; TR = 2,000 ms; FA = 90°; FOV = 220 mm × 220 mm; matrix size = 64 × 64; ST = 3 mm, slice gap = 1 mm; 35 staggered axial slices; voxel size = 3 mm × 3 mm × 3 mm; 185 volumes; and a scan time of approximately 10 min.

### GMV analysis

The 3D T1-weighted structural images were processed using the VBM8 toolbox^1^ implemented in Statistical Parametric Mapping software (SPM8).^2^ First, all structural images were segmented into gray matter, white matter, and cerebrospinal fluid density maps using a standard segmentation model. After the initial affine registration of the gray matter density map into the Montreal Neurological Institute (MNI) space, the gray matter density images were nonlinearly warped using the diffeomorphic anatomical registration through the exponentiated Lie algebra (DARTEL) technique (28). They were then resampled to a voxel size of 1.5 mm × 1.5 mm × 1.5 mm. The GMV map was obtained by multiplying the gray matter density map with the nonlinear determinants derived from the spatial normalization step. Finally, the resultant GMV images were smoothed using the full-width at the half maximum parameter of the Gaussian kernel with dimensions of 8 mm × 8 mm × 8 mm.

## Statistical analysis

Statistical descriptive analyzes of demographic and behavioral data were conducted using SPSS software (version 23.0; SPSS; Chicago, IL, United States). The one-sample Kolmogorov–Smirnov test was used to test the normality of continuous variable data, and the measurement data conforming to a normal distribution were expressed as (x ± s). One-way ANOVA and chi-square tests were used to compare the demographic characteristics and clinical psychological scale scores of the three groups. Pearson correlation analysis was used for correlation analysis between clinical scales, and statistical significance was set at *p* < 0.05.

### GMV data analysis

For the images obtained after VBM statistical analysis, a one-way ANOVA between groups was performed using SPM12 software, with age, sex, and education as covariates. The data from three groups of participants were compared using a one-way ANOVA, with *p* < 0.05 (corrected for multiple comparisons) considered to be a significant difference [using the family-wise error (FWE) method for multiple comparisons]. The GMV of all participants was analyzed based on Pearson’s correlation with their clinical assessment scales at a statistical level of *p* < 0.05. Differences in brain regions of interest (ROI) were extracted from the three groups of participants.

### General linear model analysis

The relationship between clinical symptoms and brain imaging parameters in patients with somatic symptoms in the depression group was explored. A general linear model designed using multiple regression in SPM12 software was used to identify any behaviorally significant correlation in voxel sizes of the GMV while controlling for sex, age, and education. Total intracranial volume (TIV) was an additional covariate for GMV. ROI-based analysis refers to the biased correlation analysis performed in SPSS while controlling for the above covariates.

## Results

### Comparison of sociodemographic and clinical information of the participants

The non-FSS group consisted of 37 individuals, including 7 male and 30 female individuals, who were 15.21 ± 1.35 years old and received education for 8.21 ± 1.35 years; the FSS group consisted of 34 individuals, including 8 male and 26 female individuals, who were 14.97 ± 1.54 years old and received education for 7.97 ± 1.46 years. The normal control group (NC group) consisted of 19 individuals, including 4 male and 15 female individuals, who were 15.91 ± 1.92 years old and received education for 8.47 ± 1.78 years. No statistically significant differences were observed between the three groups in terms of sex, age, and education (*p* > 0.05), and there was no statistically significant difference in disease duration between the two depressive subgroups (*p* > 0.05).

The total scores on the HAMD-17, CSI, and TAS were higher in the non-FSS and FSS groups than in the NC group, and the scores were greater in the FSS group than in the non-FSS group (*p* < 0.05), as shown in Table 1.

### Comparative GMV in the three groups

**Figure 1:**
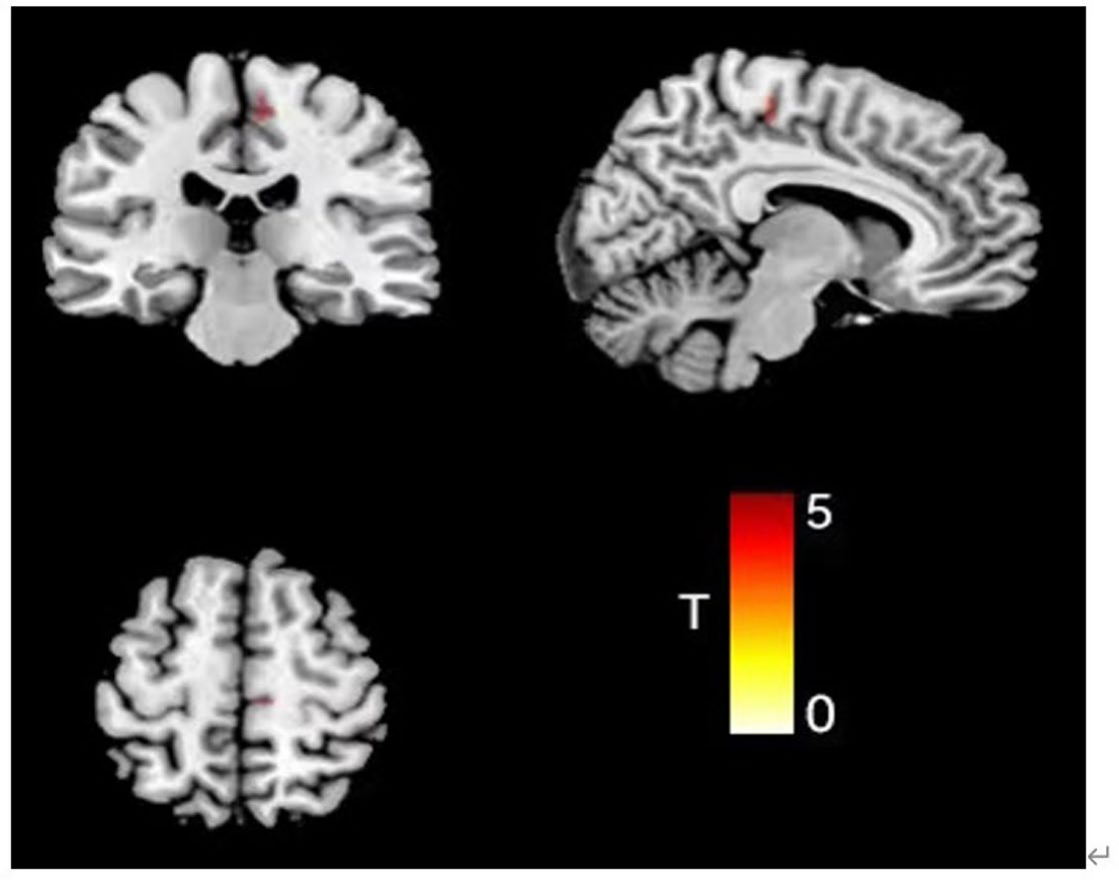
Comparison results of VBM images in the right supplementary exercisearea of the three groups of subjects (color bar represents *t* value).

After removing the effects of differences in covariates (sex, age, education, and TIV), there was a statistically significant difference between the three groups in the Supp Motor Area-R GMV (mass size: 40 voxels, peak MNI coordinates x/y/z: 7.5/−25.5/55.5, peak T: 19.506, F: 21.087, *p* < 0.001), which was greater in both the FSS (0.49 + 0.16) and non-FSS groups (0.62 + 0.17) than in the NC group (0.32 + 0.11) based on *post hoc* multiple testing. In terms of depression subgroup comparison, the GMV was greater in the non-FSS group than in the FSS group (Figure 1; Table 2).

**Table 2:** Comparison of GMV in the right supplementary motor area among the three groups.

	HC (*n* = 19)	Non-FSS (*n* = 37)	FSS (*n* = 34)	*F*	*P*
GMV	0.32 + 0.11^ab^	0.62 + 0.17^c^	0.49 + 0.16	21.087	<0.001

^a^Was compared with the group without FSS, *p* < 0.01; ^b^Was compared with the group with FSS, *p* < 0.01; ^c^Was compared with the FSS group, *p* < 0.01.

### Correlation between brain ROI and clinical symptom scales

The Supp Motor Area-R was used as the brain ROI in the depression subgroup comparison, and correlation analysis between GMV and the scores of clinical symptom scales (HAMD-17, CSI, and TAS) of the participants in the two groups were extracted. The results showed that the GMV of the Supp Motor Area-R was negatively correlated with the total CSI score in the FSS group (*r* = −0.376, *p* < 0.05), but there was no significant correlation (*p* > 0.05) with the scores of HMAD-17 and TAS. There was no significant correlation between the scores of the above-mentioned scales in the non-FSS group (*p* > 0.05; Table 3).

**Table 3:** Correlation analysis of GMV in the supplementary exercise area and clinical scale in the depression group with and without FSS.

	Non-FSS组		FSS组	
	*r*	*p*	*r*	*p*
HAMD_17_	−0.338	0.051	0.303	0.082
CSI	−0.376	0.029	0.263	0.134
CTQ	−0.302	0.099	0.077	0.679
TAS	−0.113	0.547	0.084	0.653

### Correlation between clinical scales

Analysis of clinical scales (HAMD-17, CSI, and TAS) in adolescents with depression showed a positive correlation between CSI and TAS scores (*r* = 0.378, *p* < 0.001).

### Correlation between GMV and clinical scales in the FSS group

**Figure 2:**
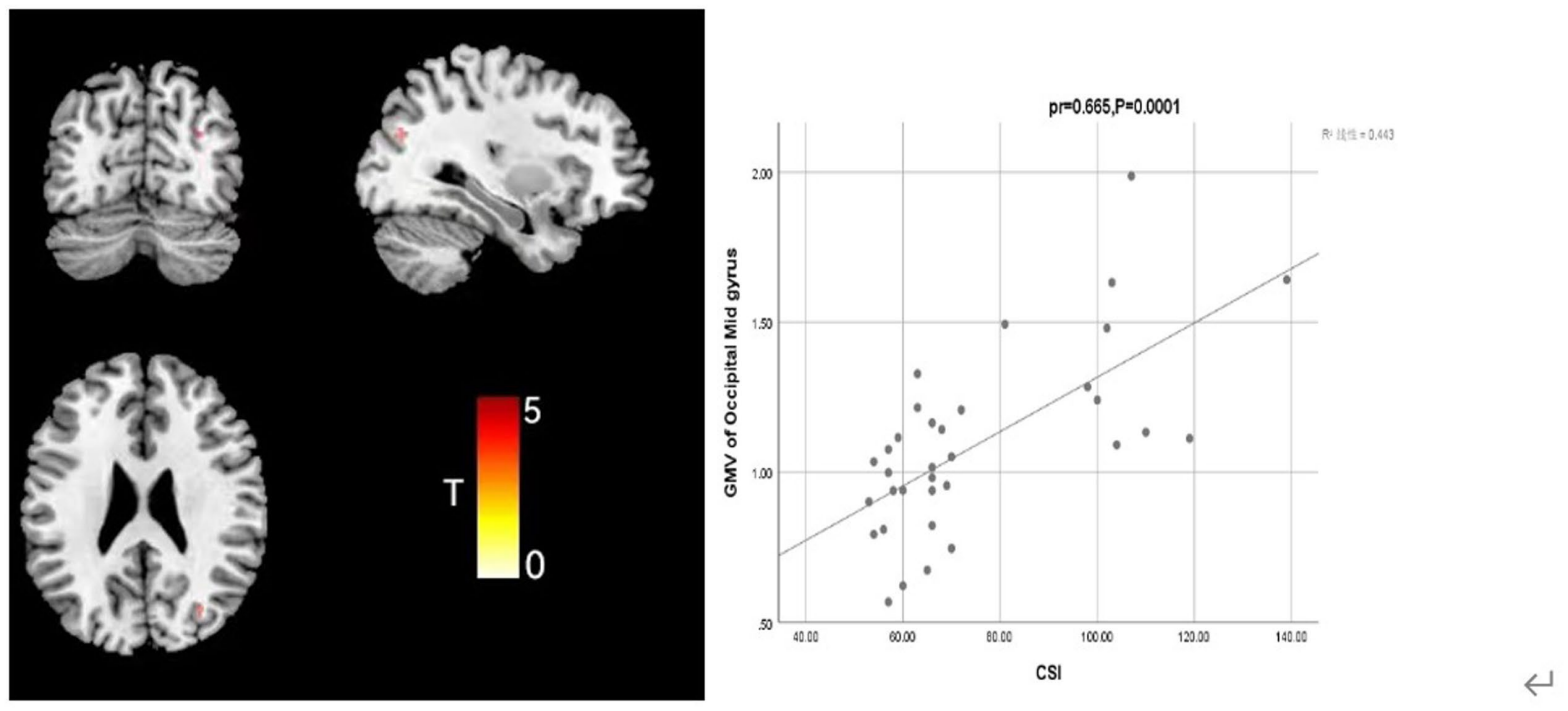
Correlation analysis between CSI score and middle occipital gyrus GMV in the depression group with somatization.

**Figure 3:**
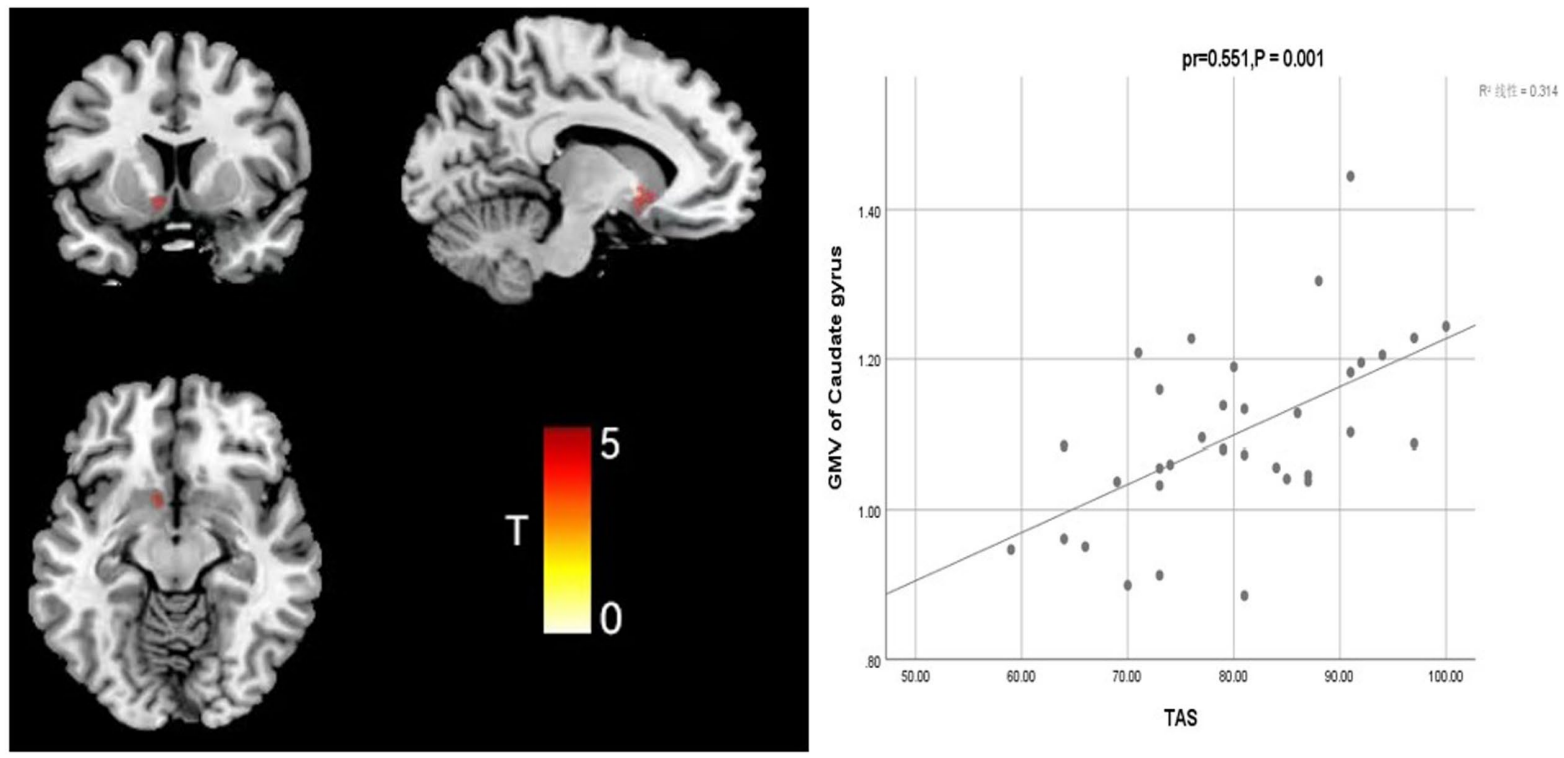
Correlation analysis between TAS score and caudate GMV in the depression group with somatization.

Further analysis of the correlation between GMV and clinical symptom scale (CSI and TAS) scores in the FSS group, when controlling for covariates such as sex, age, education, and TIV, showed that CSI score was positively correlated with GMV in the middle occipital gyrus (Occipital Mid; mass size: 31 voxels, peak MNI coordinates x/y/z: 31.5/−72/25.5, peak T: 3.41, *pr* = 0.665, *p* = 0.0001) and that TAS score was positively correlated with the GMV in the caudate nucleus (clump size: 153 voxels, peak MNI coordinates x/y/z: −7.5/16.5/−6, peak T: 5.768, *pr* = 0.551, *p* = 0.001; Figures 2, 3).

## Discussion

We found that the two adolescent depression subgroups (FSS and non-FSS groups) had greater GMV in the Supp Motor Area-R than the normal group, suggesting an increase in GMV of the Supp Motor Area-R in patients with adolescent depressive disorder relative to that of the healthy control group. Moreover, GMV was greater in the non-FSS group than in the FSS group. Previous studies have shown a reduction in GMV of the left hippocampal and cingulate gyri and thalamus and a decrease in gray matter concentration of the right inferior frontal and medial gyri in adults with depression compared to that in healthy controls (29). Compared to healthy participants, adolescents with depression had reduced GMV of the left middle temporal, right superior temporal, right middle temporal, and left superior frontal gyri and left and right precunei (30). This finding suggests that patients with depressive symptoms tend to have reduced GMV. However, in a study conducted by Shen Zonglin et al. 101 unmedicated adults with depression having their first depressive episode were divided into early-onset (18–29 years), mid-onset (30–44 years), and late-onset (45–60 years) groups, and corresponding healthy controls according to age were included. They found that GMV of the left thalamus, right lingual gyrus, cuneus, and cerebellum were reduced in the early-onset group compared with that in the young control group, while the GMV of the right middle frontal gyrus was reduced in the mid-onset group compared with that in the young control group. In the middle-onset group, the GMV of the right middle frontal gyrus decreased compared with that in the middle-aged control group. In the late-onset group, the GMV of the right middle temporal, right lingual, and left cingulate gyri and bilateral cerebellum decreased compared with that in the old-aged control group; moreover, the GMV of the right amygdala was greater in this group than in the old-aged control group (31). Increases in GMV of the medial prefrontal cortex and insular cortex, as well as the anterior hippocampus, were found in the adolescent-onset depression group relative to that in those with adult-onset depressive disorder (21). It is suggested that whole-brain GMV changes are inconsistent in adult patients with depression with different ages of onset and that different pathophysiological mechanisms may exist in these patients (21, 31).

In this study, not only was there a difference between the FSS and non-FSS depression groups in terms of Supp Motor Area-R GMV, and it was also negatively correlated with the CSI score (*r* = −0.376, *p* < 0.05) but not with the HAMD-17 score. In the FSS group, a positive correlation was found between the CSI score and GMV of the Occipital Mid. A study published in 2021 showed significantly lower regional homogeneity (ReHo)and amplitude of low-frequency fluctuation(ALFF)values in the bilateral precentral, bilateral postcentral, and left paracentral gyri in the somatic MDD group than in the depression-only group, and correlation analyzes showed that ReHo and ALFF values in these abnormal regions were negatively correlated with the severity of somatic and depressive symptoms (32). In a study of other clinical symptoms of depression, the volume of the left insula and the left triangular part of the inferior frontal gyrus was found to be reduced in the depressed group without anxiety compared with that in the normal group, and the depressed group with anxiety showed greater GMV in the midbrain, medial prefrontal cortex, and primary motor/somatosensory cortex and greater frontal GMV than in the group without anxiety (33). In depressed patients with psychotic symptoms, anterior insular GMV was not associated with depression severity, but left anterior insular GMV was significantly associated with total and positive symptom severity of psychosis (34). A 2016 study found differences in brain area activity when reflecting on pictures of different faces in patients with different clinical subtypes of reality-depersonalization disorder. Results showed that the differentiated regions for depressive symptoms were the right occipital nucleus (happy) and the left amygdala (sad); for somatic symptoms, they were in the right temporal cap (happy) and the ventral striatum (sad); and for the state of anxiety, the differentiated regions were the left inferior frontal gyrus (happy) and the parahippocampal gyrus (sad). These results suggest that each clinical symptom may involve a functional brain system (35). In patients with somatization disorder, the severity of somatization was found to correlate with patients’ cingulate and sub-frontal-occipital tract anisotropy score values, suggesting that somatic symptoms may be associated with altered white matter integrity in the cingulate, sub-frontal-occipital tract, anterior thalamic radiation, and corticospinal tract and not just with GMV (36). These studies suggest that depressive and somatic symptoms in adolescent depressive disorder may affect different brain regions or result from the interaction of different brain regions. The Supp Motor Area-R and Occipital Mid may be the brain regions associated with somatic symptoms in adolescent depressive disorder.

Alexithymia is a relatively stable personality trait that is often present in patients with certain psychiatric disorders involving difficulty in identifying emotions or describing and communicating with others, lack of emotion-related fantasies, and extroverted thinking (37). We found that both FSS and non-FSS groups had higher TAS scores than the NC group (*p* < 0.05) and that the CSI score was positively correlated with TAS scores (*r* = 0.378, *p* < 0.001). TAS scores were positively correlated with caudate nucleus GMV in the FSS group. Previous studies have suggested that patients with alexithymia disorder are unable to recognize their emotional disorders and express their emotions; therefore, they translate their bad emotions into somatic discomfort (38). Farah et al. showed that the amygdala is a brain region associated with alexithymia disorder, and increased volume of the right amygdala may contribute to the development of narrative disorder (39). A meta-analysis by Donges et al. which included 22 alexithymia disorders, revealed deficits in the automatic processing of emotional stimuli at behavioral and neurobiological levels in patients with alexithymia disorders, showing that reduced levels of spontaneous insula activation in patients with alexithymia disorders attenuated their emotional responses and recognition (40). Alterations in brain structure or function cause individuals to deliberately avoid emotion-related stimuli, and reduced emotional activity leads to reduced function in emotion-related brain areas such that alterations in brain structure or function result in narrative affective disorders (41). Previous studies have found that caudate nucleus GMV is associated with insomnia symptoms and negative perceptions of depression (42). A 2015 study analyzed the dimensions and subtypes of alexithymia impairment by measuring gray and white matter volumes using voxel-based morphometry, with Type I (impairment in both dimensions) and Type II (impairment in the cognitive dimension) alexithymia impairment characterized by reduced volume in the left amygdala and thalamus, respectively. Cognitive dimension impairment was associated with reduced volumes in the right amygdala, left posterior insula, precuneus, caudate nucleus, hippocampus, and parahippocampus (43). Type III (impairment of the affective dimension but not of the cognitive dimension) alexithymia impairment is characterized only by the reduced volume of the middle cingulate cortex. The affective dimension impairment is further characterized by the increased volume of the anterior cingulate cortex, which provides evidence for distinct neuroanatomical representations of the different narrative impairment subtypes. In this study, CSI scores were found to be correlated with the TAS scores in adolescent depressive disorder and were positively correlated with the GMV of the caudate nucleus, suggesting a correlation between somatic symptoms and alexithymia disorder in patients with adolescent depressive disorder, but further differentiation of the correlation between different types of alexithymia disorder and specific brain regions is needed.

This study has some limitations. First, we studied healthy participants and patients with depression (with and without somatic symptoms), but somatic symptoms are a clinical symptom cluster, and the study did not distinguish between different somatic symptoms (pain, gastrointestinal symptoms, pseudoaneurysms, etc.). Somatic symptom clusters may have heterogeneity affecting the study structure, and further sample size expansion is needed in the future to compare brain imaging and cellular characteristics of different somatic symptom factors and correlations of inflammatory factors. Second, causality cannot be inferred from this cross-sectional design, especially when exploring interactions between psychology and brain imaging; longitudinal studies or studies with expanded sample sizes targeting interventions to improve depression and somatic symptoms need to be conducted in the future. The introduction of research methods such as mediation analysis may be needed to determine the direction of causality. The third limitation of the study is that patients had different disease duration, number of episodes, and antidepressant treatments involving different medication doses and types, physical therapy (transcranial magnetic stimulation), and psychotherapy. These differences in disease duration and treatment modalities may have affected the study results. Future studies on unmedicated patients with the first episode are needed to validate the preliminary results of this study.

In conclusion, we found that the HAMD-17 and TAS scores in the FSS group were greater than those in the non-FSS group and that the CSI score was positively correlated with HAMD-17 and TAS scores. The GMV of the Supp Motor Area-R was greater in the depression subgroups (FSS and non-FSS groups) than in the NC group; moreover, GMV was greater in the non-FSS group than in the FSS group. In the FSS group, the CSI score was negatively correlated with the Supp Motor Area-R and Occipital Mid GMV, while TAS scores were positively correlated with the GMV of the caudate nucleus. Therefore, somatic symptom impairment in adolescent patients with depression correlates with GMVs in different brain regions.

## Data availability statement

The raw data supporting the conclusions of this article will be made available by the authors, without undue reservation.

## Ethics statement

This study was approved by the Ethics Committee of the Anhui Mental Health Center, Hefei Fourth People’s Hospital (no. HSY-IRB-PJ-XJJ-ZH003). After a complete description of the study was provided, all the participants’ legal guardians provided written informed consent.

## Author contributions

HL and HZ: study design. DM, PG, SH, and RT: collection, analyzes, and interpretation of data. DM: drafting the first version of the manuscript. HL and HZ: critical revision of the manuscript. All authors contributed to the article and approved the submitted version.

## Funding

The study was supported by the Anhui Provincial Department of Science and Technology (no. 1804 h08020251) and National Key Research and Development Program (no. 2018YFC1314300).

## Conflict of interest

The authors declare that the research was conducted in the absence of any commercial or financial relationships that could be construed as a potential conflict of interest.

## Publisher’s note

All claims expressed in this article are solely those of the authors and do not necessarily represent those of their affiliated organizations, or those of the publisher, the editors and the reviewers. Any product that may be evaluated in this article, or claim that may be made by its manufacturer, is not guaranteed or endorsed by the publisher.

## Footnotes

^1^ http://dbm.neuro.uni-jena.de/vbm.html

^2^ http://www.fil.ion.ucl.ac.uk/spm

## References

[1] Huang Y Wang Y Wang H Liu Z Yu X Yan J. Prevalence of mental disorders in China: a cross-sectional epidemiological study. Lancet Psychiatry. (2019) 6:211–24. doi: 10.1016/S2215-0366(18)30511-X, PMID: 30792114

[2] Li F Cui Y Li Y Guo L Ke X Liu J. Prevalence of mental disorders in school children and adolescents in China: diagnostic data from detailed clinical assessments of 17,524 individuals. J Child Psychol Psychiatry. (2022) 63:34–46. doi: 10.1111/jcpp.13445, PMID: 34019305

[3] Sacchet MD Ho TC Connolly CG Tymofiyeva O Lewinn KZ Han LK. Large-scale Hypoconnectivity between resting-state functional networks in Unmedicated adolescent major depressive disorder. Neuropsychopharmacology. (2016) 41:2951–60. doi: 10.1038/npp 27238621 PMC5061890

[4] Avenevoli S Swendsen J He JP Burstein M Merikangas KR. Major depression in the national comorbidity survey-adolescent supplement: prevalence, correlates, and treatment. J Am Acad Child Adolesc Psychiatry. (2015) 54:37. doi: 10.1016/j.jaac.2014.10.010, PMID: 25524788 PMC4408277

[5] Kop WJ. Somatic depressive symptoms, vital exhaustion, and fatigue: divergent validity of overlapping constructs. Psychosom Med. (2012) 74:442–5. doi: 10.1097/PSY.0b013e31825f30c7 22685237

[6] Simon GE VonKorff M Piccinelli M Fullerton C Ormel J. An international study of the relation between somatic symptoms and depression. N Engl J Med. (1999) 341:1329–35. doi: 10.1056/NEJM199910283411801 10536124

[7] Zhou X Peng Y Zhu X Dere J Chentsova-Dutton YE Ryder AG. From culture to symptom: testing a structural model of “Chinese somatization”. Transcult Psychiatry. (2016) 53:3–23. doi: 10.1177/1363461515589708 26076689

[8] Yingying D Min Z Gu L Xiaoyan K Kangkan GCH Bin Xu. Children and adolescents’ functional somatization symptoms and related factors in urban area Nanjing. China. J Health Psychol. (2018) 26:1441–5. doi: 10.13342/j.cnki.cjhp.2018.10.001

[9] Beck JE. A developmental perspective on functional somatic symptoms. J Pediatr Psychol. (2007) 33:547–62. doi: 10.1093/jpepsy/jsm113, PMID: 18056142

[10] Campo JV. Annual research review: functional somatic symptoms and associated anxiety and depression--developmental psychopathology in pediatric practice. J Child Psychol Psychiatry. (2012) 53:575–92. doi: 10.1111/j.1469-7610.2012.02535, PMID: 22404290

[11] Bohman H Jonsson U Päären A von Knorring L Olsson G von Knorring AL. Prognostic significance of functional somatic symptoms in adolescence: a 15-year community-based follow-up study of adolescents with depression compared with healthy peers. BMC Psychiatry. (2012) 12:90. doi: 10.1186/1471-244X-12-90, PMID: 22839681 PMC3439696

[12] Bekhuis E Boschloo L Rosmalen JG de Boer MK Schoevers RA. The impact of somatic symptoms on the course of major depressive disorder. J Affect Disord. (2016) 205:112–8. doi: 10.1016/j.jad.2016.06.030, PMID: 27428090

[13] Klengel T Heck A Pfister H Brückl T Hennings JM Menke A. Somatization in major depression--clinical features and genetic associations. Acta Psychiatr Scand. (2011) 124:317–28. doi: 10.1111/j.1600-0447.2011.01743.x, PMID: 21838737

[14] Taycan O Ozdemir A Erdogan Taycan S. Alexithymia and somatization in depressed patients: the role of the type of somatic symptom attribution. Noro Psikiyatr Ars. (2017) 54:99–104. doi: 10.5152/npa.2016.12385, PMID: 28680305 PMC5491676

[15] Barrett LF Quigley KS Hamilton P. An active inference theory of allostasis and interoception in depression. Philos Trans R Soc Lond Ser B Biol Sci. (2016) 371:20160011. doi: 10.1098/rstb.2016.0011 28080969 PMC5062100

[16] Harshaw C. Interoceptive dysfunction: toward an integrated framework for understanding somatic and affective disturbance in depression. Psychol Bull. (2015) 141:311–63. doi: 10.1037/a0038101 25365763 PMC4346391

[17] López-Solà M Pujol J Hernández-Ribas R Harrison BJ Contreras-Rodríguez O Soriano-Mas C. Effects of duloxetine treatment on brain response to painful stimulation in major depressive disorder. Neuropsychopharmacology. (2010) 35:2305–17. doi: 10.1038/npp.2010.108, PMID: 20668437 PMC3055320

[18] Persson J Larsson A Reuter-Lorenz PA. Imaging fatigue of interference control reveals the neural basis of executive resource depletion. J Cogn Neurosci. (2013) 25:338–51. doi: 10.1162/jocn_a_00321 23163416

[19] Tang LW Zheng H Chen L Zhou SY Huang WJ Li Y. Gray matter volumes in patients with chronic fatigue syndrome. Evid Based Complement Altern Med. (2015) 2015:380615. doi: 10.1155/2015/380615, PMID: 25792998 PMC4352504

[20] Besteher B Gaser C Langbein K Dietzek M Sauer H Nenadić I. Effects of subclinical depression, anxiety and somatization on brain structure in healthy subjects. J Affect Disord. (2017) 215:111–7. doi: 10.1016/j.jad.2017.03.039, PMID: 28319687

[21] Blank TS Meyer BM Wieser MK Rabl U Schögl P Pezawas L. Brain morphometry and connectivity differs between adolescent- and adult-onset major depressive disorder. Depress Anxiety. (2022) 39:387–96. doi: 10.1002/da.23254, PMID: 35421280 PMC9323432

[22] Van Geelen SM Rydelius PA Hagquist C. Somatic symptoms and psychological concerns in a general adolescent population: exploring the relevance of DSM-5 somatic symptom disorder. J Psychosom Res. (2015) 79:251–8. doi: 10.1016/j.jpsychores.2015.07.012, PMID: 26297569

[23] Romero-Acosta K Canals J Hernández-Martínez C Penelo E Zolog TC Domènech-Llaberia E. Age and gender differences of somatic symptoms in children and adolescents. J Ment Health. (2013) 22:33–41. doi: 10.3109/09638237.2012.734655, PMID: 23343045

[24] Ho TC Sacchet MD Connolly CG Margulies DS Tymofiyeva O Paulus MP. Inflexible functional connectivity of the dorsal anterior cingulate cortex in adolescent major depressive disorder. Neuropsychopharmacology. (2017) 42:2434–45. doi: 10.1038/npp.2017.103, PMID: 28553837 PMC5645733

[25] Zheng YP Zhao JP Phillips M Liu J Cai M Sun S. Validity and reliability of the Chinese Hamilton depression rating scale. Br J Psychiatry. (1988) 152:660–4. doi: 10.1192/bjp.152.5.660 3167442

[26] Yuanyuan Z Guo L Junfeng H Baihui G Xumei W. Revising, eliability and validity of Chinese version Children’s somatization inventory. J Int Psychiatry. (2015) 42:12–5.

[27] Jinyao Y Shuqiao Y Xiongzhao Z. The Chinese version of the TAS-20:reliability and validity. Chin J Health Psychol. (2003) 17:763–7. doi: 10.1037/0735-7028.26.4.427

[28] Ashburner J. A fast diffeomorphic image registration algorithm. NeuroImage. (2007) 2007:95–113. doi: 10.1016/j.neuroimage.2007.07.007 17761438

[29] Vasic N Walter H Hose A Wolf RC. Gray matter reduction associated with psychopathology and cognitive dysfunction in unipolar depression: a voxel-based morphometry study. J Affect Disord. (2008) 109:107–16. doi: 10.1016/j.jad.2007.11.011, PMID: 18191459

[30] Li X Chen X Yu R Dai L Ai M Huang Q. Changes in gray matter volume following electroconvulsive therapy in adolescent depression with suicidal ideation: a longitudinal structural magnetic resonance imaging study. Front Psych. (2022) 13:944520. doi: 10.3389/fpsyt.2022.944520, PMID: 36245857 PMC9559807

[31] Shen Z Cheng Y Yang S Dai N Ye J Liu X. Grey matter volume in first-episode adult major depressive disorder with different age-onset. Chin J Psychiatry. (2017) 50:193–200. doi: 10.1016/j.nicl.2016.08.016 PMC5026687 27668175

[32] Liu P Tu H Zhang A Yang C Liu Z Lei L. Brain functional alterations in MDD patients with somatic symptoms: a resting-state fMRI study. J Affect Disord. (2021) 295:788–96. doi: 10.1016/j.jad.2021.08.143, PMID: 34517253

[33] Qi H Ning Y Li J Guo S Chi M Gao M. Gray matter volume abnormalities in depressive patients with and without anxiety disorders. Medicine (Baltimore). (2014) 93:e345. doi: 10.1097/MD.0000000000000345, PMID: 25546687 PMC4602623

[34] Cohen JD Nichols T Keller J Gomez RG Schatzberg AF Reiss AL. Insular cortex abnormalities in psychotic major depression: relationship to gender and psychotic symptoms. Neurosci Res. (2013) 75:331–9. doi: 10.1016/j.neures.2013.02.005, PMID: 23471015 PMC3662543

[35] Lemche E Surguladze SA Brammer MJ Phillips ML Sierra M David AS. Dissociable brain correlates for depression, anxiety, dissociation, and somatization in depersonalization-derealization disorder. CNS Spectr. (2016) 21:35–42. doi: 10.1017/S1092852913000588, PMID: 24059962

[36] Zhang J Jiang M Yao D Dai Y Long L Yu M. Alterations in white matter integrity in first-episode, treatment-naive patients with somatization disorder. Neurosci Lett. (2015) 599:102–8. doi: 10.1016/j.neulet.2015.05.037 26003450

[37] Taylor GJ. Recent developments in alexithymia theory and research. Can J Psychiatry. (2000) 45:134–42. doi: 10.1177/070674370004500203 10742872

[38] Saariaho AS Saariaho TH Mattila AK Karukivi MR Joukamaa MI. Alexithymia and depression in a chronic pain patient sample. Gen Hosp Psychiatry. (2013) 35:239–45. doi: 10.1016/j.genhosppsych.2012.11.011, PMID: 23333032

[39] Farah T Ling S Raine A Yang Y Schug R. Alexithymia and reactive aggression: the role of the amygdala. Psychiatry Res Neuroimag. (2018) 281:85–91. doi: 10.1016/j.pscychresns.2018.09.003, PMID: 30273792 PMC6226305

[40] Donges US Suslow T. Alexithymia and automatic processing of emotional stimuli: a systematic review. Rev Neurosci. (2017) 28:247–64. doi: 10.1515/revneuro-2016-0049, PMID: 28099136

[41] Förster K Enneking V Dohm K Redlich R Meinert S Geisler AI. Brain structural correlates of alexithymia in patients with major depressive disorder. J Psychiatry Neurosci. (2020) 45:117–24. doi: 10.1503/jpn.190044, PMID: 31603638 PMC7828911

[42] Ho TC Teresi GI Ojha A Walker JC Kirshenbaum JS Singh MK. Smaller caudate gray matter volume is associated with greater implicit suicidal ideation in depressed adolescents. J Affect Disord. (2021) 278:650–7. doi: 10.1016/j.jad.2020.09.046, PMID: 33039875 PMC9386733

[43] Goerlich-Dobre KS Votinov M Habel U Pripfl J Lamm C. Neuroanatomical profiles of alexithymia dimensions and subtypes. Hum Brain Mapp. (2015) 36:3805–18. doi: 10.1002/hbm.22879 26094609 PMC6869665

